# MultiPhATE2: code for functional annotation and comparison of phage genomes

**DOI:** 10.1093/g3journal/jkab074

**Published:** 2021-03-17

**Authors:** Carol L Ecale Zhou, Jeffrey Kimbrel, Robert Edwards, Katelyn McNair, Brian A Souza, Stephanie Malfatti

**Affiliations:** 1 Global Security Computing Applications, Lawrence Livermore National Laboratory, Livermore, CA 94550, USA; 2 Biosciences & Biotechnology Research Division, Lawrence Livermore National Laboratory, Livermore, CA 94550, USA; 3 Computational Sciences Research Center, San Diego State University, San Diego, CA 92182, USA; 4 Department of Biology, San Diego State University, San Diego, CA 92182, USA; 5 Viral Information Institute, San Diego State University, San Diego, CA 92182, USA; 6 College of Science and Engineering, Flinders University, Bedford Park, SA 5048, Australia

**Keywords:** gene prediction, genome annotation, comparative genomics, bacteriophage, phage, bioinformatics tool

## Abstract

To address a need for improved tools for annotation and comparative genomics of bacteriophage genomes, we developed multiPhATE2. As an extension of multiPhATE, a functional annotation code released previously, multiPhATE2 performs gene finding using multiple algorithms, compares the results of the algorithms, performs functional annotation of coding sequences, and incorporates additional search algorithms and databases to extend the search space of the original code. MultiPhATE2 performs gene matching among sets of closely related bacteriophage genomes, and uses multiprocessing to speed computations. MultiPhATE2 can be re-started at multiple points within the workflow to allow the user to examine intermediate results and adjust the subsequent computations accordingly. In addition, multiPhATE2 accommodates custom gene calls and sequence databases, again adding flexibility. MultiPhATE2 was implemented in Python 3.7 and runs as a command-line code under Linux or MAC operating systems. Full documentation is provided as a README file and a Wiki website.

## Introduction

As the era of reliable antibacterial treatment draws to a close, bacteriophage (phage) therapy is gaining ground in Western countries as an alternative treatment for antibiotic resistant and chronic recalcitrant bacterial infections, with several clinical trials having recently been initiated (Furfaro *et al.* 2018; Altamirano and Barr 2019; Górski *et al.* 2020; Voelker 2019; Duplessis and Biswas 2020; Pires *et al.* 2020). These efforts depend on reliable, actionable biological information, much of which is generated today using bioinformatics tools for analyzing the increasingly large quantities of genomic sequencing data. Moreover, it is essential that the genomes are sequenced and accurately annotated to avoid introducing unwanted genes (such as toxins or antibiotic resistance genes) into the patient (Luong et al. 2020).

To support these medical research efforts, open-source tools for phage genome annotation are prerequisite. Although next-generation sequencing efforts have generated increasingly large quantities of phage genomic data (Russell and Hatfull 2017; Carrol *et al.* 2018), there still remains an urgent need for tools that enable annotation and evaluation of phage genomic data and that inform research efforts toward developing novel phage therapies and therapeutics based on phage products (Yang *et al.* 2014; Górski *et al.* 2020). A number of advanced bioinformatics tools and computational systems have been developed for evaluation of phage sequence, including detection of prophage sequences within bacterial genomes (Akhter *et al.* 2012; Reis-Cunha *et al.* 2019; and others), evaluation of phage sequence for therapeutic goals (Philipson *et al.* 2018), and working with phage metagenomics data (Kieft *et al.* 2020). Several microbial genome annotation tools have been used for annotation of phage genomes, including EDGE (Li *et al.* 2017), PROKKA (Seemann 2014), DFAST (Tanizawa *et al.* 2019), RAST (Aziz *et al.* 2008), and PATRIC (Davis *et al.* 2020). Collectively, these tools offer a broad array of capabilities ranging from genome assembly to gene prediction to functional annotation and standardized formatting of data for submission to public databases. For example, EDGE is a bioinformatics platform that enables comprehensive processing of metagenomic data, including assembly and annotation, taxonomic classification, phylogenetic analysis, and primer analysis. DFAST and PROKKA perform protein and noncoding gene predictions, functional annotation targeted at prokaryotes, and are largely intended for rapid analysis and submission to databases. PATRIC offers comprehensive genome analysis using vast data sets comprising curated data for pathogen species and RAST annotation tools “under the hood.” It is important to note that these tools are primarily aimed at evaluation of bacterial and/or archaeal genome sequence.

As of this writing, we are not aware of any comprehensive, open-source annotation system that is tailored for phage annotation. We developed multiPhATE (Ecale Zhou *et al.* 2019) to address the need for a downloadable, command-line annotation tool suited for use by phage research laboratories with few or no bioinformatics specialists on staff, and limited resources dedicated to constructing annotation and comparative genomics pipelines from individual tools and databases. Here, we describe updates that we have made to the original multiPhATE code, resulting in an even more versatile and flexible tool. Updates to be found in multiPhATE2 include additional database search algorithms and supported databases, parallel processing to speed computations, controls that add flexibility to the workflow, and new code for comparing across related genomes.

## Methods

### MultiPhATE2 system overview

**Figure 1:**
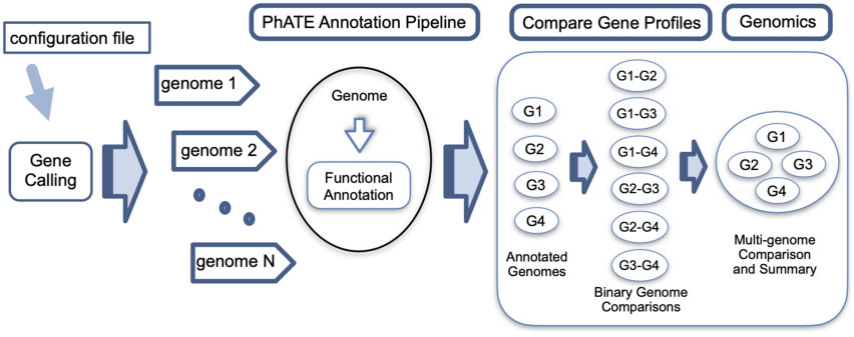
Overview of multiPhATE2 system and workflow. User-specified configurations (configuration file) are input to the multiPhATE2 system, which invokes four subsystems: Gene Calling, the PhATE annotation pipeline, Compare Gene Profiles, which performs binary genome-to-genome comparisons of genes and proteins, and Genomics, which consolidates binary comparisons into gene–gene and protein–protein correspondences among all input genomes.

MultiPhATE2 comprises a comprehensive, high-throughput functional annotation and genome comparison system for analysis of newly sequenced phage genomes. An accounting of multiPhATE2 features, and a comparison to those of the original multiPhATE code can be found in the multiPhATE2 GitHub wiki (see “What are the differences between the original multiPhATE code and multiPhATE2?”; URL is provided below). MultiPhATE2 performs gene finding followed by computational functional annotation of user-specified phage genomes, then performs gene-by-gene comparisons among the genomes. A system driver script takes a single argument consisting of a configuration file, then invokes up to four computational subsystems: the Gene Calling and PhATE annotation subsystems are run for each genome, and, if two or more genomes are specified by the user, multiPhATE will identify corresponding genes among the genomes using the Compare Gene Profiles (Tkavc *et al.* 2017) and Genomics subsystems (Figure 1).

**Figure 2:**
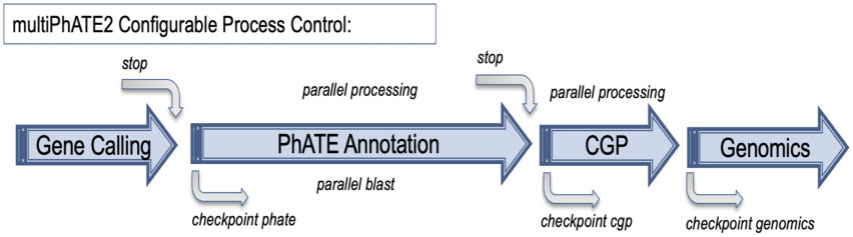
System overview and configurable process control features of multiPhATE2. Large blue arrows: multiPhATE2 subsystems; CGP = Compare Gene Profiles; curved grey arrows: process controls (stop = stopping point; checkpoint = point at which processing may be restarted); “parallel processing” indicates multiprocessing applied to functional annotation of input genomes and binary genome-to-genome comparisons; “parallel blast” indicates multithreading option provided by BLAST+.

Recognizing a compelling need for flexible and rapid computations, we have included process control features within the multiPhATE2 workflow (Figure 2). We believe that multiPhATE2 scales well in performing multi-genome annotation and comparison, while offering a flexible toolset for tailoring analyses according to the user’s needs.

### Process control

Parallel processing is optionally applied at several stages of computation (Figure 2). Furthermore, the user may stop or re-start computations at several points within the workflow. These options may be selected in the multiPhATE2 system configuration file. Specifically,

1. Parallel processing is applied in the PhATE and Compare Gene Profiles subsystems. Each genome input to PhATE may be processed as a separate, parallel process, and each binary genome comparison in the Compare Gene Profiles subsystem may be processed likewise in parallel.
2. The user may specify the desired number of threads with which to invoke Blastp (Camacho *et al.* 2009) in the PhATE subsystem.
3. The user may opt to process the multiPhATE2 code through the Gene Finding subsystem or the PhATE subsystem and stop at either point.
4. The user may opt to re-start processing at three points in the multiPhATE2 system. Checkpoints (re-starting points) may be selected at the beginning of the PhATE, Compare Gene Profiles, or Genomics subsystem processing.

### Input

MultiPhATE2 is invoked using a single input parameter consisting of a configuration file, as follows: $python multiPhate.py multiphate.config. The configuration file allows the user to specify (a) genomes to be processed, (b) gene finder(s) to be used, (c) PhATE annotation search algorithms and databases, (d) blast cutoffs, (e) locations of databases, (f) Compare Gene Profiles matching cutoff, (g) PhATE, Compare Gene Profiles, and Blast+ multiprocessing, (h) stopping points, (i) checkpoints, and (j) console messaging verbosity. Concise instructions for creating a multiphate configuration file are provided in the project README, the project wiki, and in the sample.multiphate.config file itself, provided with the multiPhATE2 distribution. Code execution can be tailored to run specific gene finders and to search for homologous sequences in specific phage- and virus-centric data sets, in addition to more generic protein data sets.

### Gene calling subsystem

**Figure 3:**
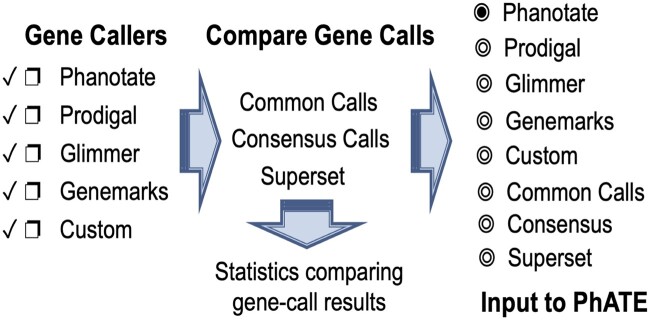
Gene callers and gene-call comparison in the Gene Calling subsystem of multiPhATE2. The user may select any or all of the supported gene callers and/or provide their own gene calls (custom). The Compare Gene Calls module computes a set of calls that are common among all selected callers (common calls), a consensus set comprising gene calls produced by at least two callers (consensus calls), and a nonredundant superset of gene calls (superset). The user may select the results of one gene caller or a super/subset for input to the PhATE subsystem.

The Gene Calling subsystem of multiPhATE was updated to include user-provided GFF-formatted custom gene calls, in addition to the already-supported Glimmer (Delcher *et a*l. 2007), GeneMarkS (Besemer *et al.* 2001), Prodigal (Hyatt *et al.* 2010), and PHANOTATE (McNair *et al.* 2019, McNair et al. 2021) gene callers, so that it is now also possible to include and compare gene calls from web-only based services, Genbank gene calls, or hand-curated gene-call data sets (see README in the project repository for format specifications). The side-by-side comparison among gene callers (Compare Gene Calls module, Figure 3) was expanded to include output data sets that either merge the results of multiple gene callers (*i.e.*, a nonredundant superset), or that recognize agreement among callers: a consensus gene-call set, comprising calls that were in agreement among two or more callers, and a common-core gene-call set representing calls that were made by all the gene callers. Any one of the gene-call outputs, custom calls, or multiPhATE-generated gene-call super/subsets may be forwarded on for PhATE functional annotation.

### PhATE annotation subsystem

**Figure 4:**
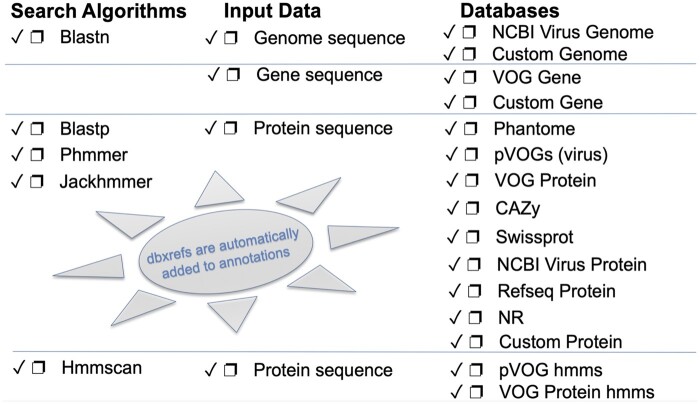
Functional annotation options supported within the PhATE subsystem of multiPhATE2. The user may select any or all of the algorithms to search any or all of the databases over genome, gene, and/or protein sequences. dbxrefs = database external references, which comprise additional information about a given database entry.

PhATE is a fully automated computational pipeline for functional annotation of phage genes within a genome sequence, and was originally written as part of multiPhATE (Ecale Zhou *et al.* 2019). Newly incorporated analyses include: blast and hmm searches for VOG gene and protein (Laffy *et al.* 2016), CAZy (Lombard *et al.* 2014), custom genome, gene, and protein databases; HMM profile searches for pVOG-hmm (Grazziotin *et al.* 2017) and VOG-protein-hmm (Laffy *et al.* 2016) databases; and new searches with phmmer and hmmscan (Johnson *et al.* 2010). Preprocessing of the pVOG, VOG, and CAZy data sets are performed in order to speed the matching of hits with associated annotations. A script is provided to facilitate downloading, preprocessing, and updating of databases that are supported in multiPhATE2 (dbPrep_getDBs.py). A full accounting of the search algorithms and databases supported by the PhATE annotation subsystem within multiPhATE2 is depicted in Figure 4.

### Comparative genomics subsystems

MultiPhATE2 accomplishes comparisons among input genomes in a two-step process whereby each genome is compared to each other, and then gene-gene and protein–protein correspondences are identified from among all of the input genomes.

The Compare Gene Profiles subsystem performs NxN reciprocal blast of the genes from each genome against the genes from every other genome provided by the user (Figure 1). The code then identifies for each gene its mutual and nonmutual (singular) best hits against corresponding genes from each of the other genomes, or reports if no corresponding gene is found. For each binary genome-to-genome comparison, hits are ordered with respect to the query genome. The Genomics subsystem inputs the binary blast result files from Compare Gene Profiles and computes genes and proteins that correspond across all the input genomes with respect to the reference genome (*i.e.*, the first genome listed). Ultimately, homology groups are generated, comprising each reference gene and its corresponding genes, plus its paralog’s corresponding genes. This analysis is also performed for protein sequences. PhATE annotations are carried through the Compare Gene Profiles and Genomics computations so that the user can readily identify gene/protein function among the identified homolog groups.

### MultiPhATE system output

Directories and files that are produced by multiPhATE2 are detailed in the README. In brief, an output subdirectory is created for each input genome to hold results of the Gene Finding and PhATE subsystems, and subdirectories are created to hold results of the Compare Gene Profiles and Genomics subsystems. A sample multiPhATE2 system main output directory with contents for the Bacteriophage P2 genome (Christie and Calendar 2016) can be found in the multiPhATE2 supplementary data repository. In summary, the following subdirectories are created:

1. Genome result directories, one for each genome processed through the Gene Calling and PhATE subsystems, including (a) results of gene calling, (b) BLAST, HMM, and PROFILE directories containing intermediate and final results of searches, (c) fasta groupings comprising query sequences with their pVOG or VOG homologs, (d) final results of side-by-side gene calling comparison (file: CGC_results.txt) and final results of functional annotation (files: phate_sequenceAnnotation_main.out/.gff).
2. CGP_RESULTS directory: Output from the Compare Gene Profiles subsystem,
3. GENOMICS_RESULTS directory: Output from the Genomics subsystem.
4. JSON directory: Contains one automatically generated JSON configuration file for each instance of PhATE annotation (*i.e.*, per genome). The JSON files convey user-specified input parameters to the PhATE subsystem and serve as a record of analyses performed.

### Data availability

MultiPhATE2 is freely available under an open-source GPL3 license at https://github.com/carolzhou/multiPhATE2. Instructions for downloading, installing, and using multiPhATE2, as well as instructions for acquiring databases and third party codes used by multiPhATE2, are found in the README file included with the distribution. Supplementary materials, which demonstrate the outputs of multiPhATE2, are available in a GitHub repository at https://github.com/carolzhou/multiPhATE2_supplementaryData/. Additionally, use cases illustrating many of the capabilities of the software, as well as a chart depicting additional features of multiPhATE2 compared to is predecessor, can be found on the project wiki pages, at https://github.com/carolzhou/multiPhATE2/wiki (or select the “Wiki” tab in the project repository). Users may report software issues on the project’s GitHub repository webpage (select the “Issues” tab) or by sending an email to multiphate@gmail.com.

## Results and discussion

MultiPhATE2 represents a significant advance in bacteriophage genome annotation in that it streamlines gene calling, functional sequence annotation, and comparative genomics for sets of newly sequenced draft or finished genomes. MultiPhATE is straight forward to install, with full instructions in the README file in the project’s github repository. Running multiPhATE2 as a command-line program taking a single argument (*i.e.*, the multiphate.config file) facilitates launching jobs comprising annotation and comparison of potentially large sets of genomes. Furthermore, built-in flexibility (Figure 2) allows the user not only to install components in a step-wise manner, but also to run and re-run each subsystem with different parameters, so that the user may determine, for example, (a) which gene caller or callers are preferred and which one may be best to carry through to functional annotation; (b) which search algorithms and databases (including custom) are most appropriate for the genomes under study; (c) which sequence identity cutoffs produce the desired stringency in terms of homolog identification or gene-gene correspondence. MultiPhATE2 offers a wide range of gene calling and sequence search algorithms and databases, including the options of providing user-defined custom gene calls and blast databases (Figures 3 and 4). Parallel processing within the PhATE and CGP subsystems (Figure 2) enables computations for large input data sets, which might not otherwise be feasible when processing in serial. Furthermore, hits to entries in each of the pVOG and VOG databases are combined with the query protein sequence to produce fasta grouping to facilitate potential follow-on analyses (beyond the scope of multiPhATE2), such as multiple sequence alignment and common motif identification. We know of no other phage-tailored genome annotation system that provides breadth and flexibility that are comparable to multiPhATE2.

## References

[jkab074-B1] Akhter S , Aziz RK, Edwards RA. 2012. PhiSpy: a novel algorithm for finding prophages in bacterial genomes that combines similarity- and composition-based strategies. Nucleic Acids Res. 40:e126. 22584627 10.1093/nar/gks406 PMC3439882

[jkab074-B2] Altamirano FLG , Barr JJ. 2019. Phage therapy in the post antibiotic era. Clin Microbiol Rev. 32:e00066-18.30651225

[jkab074-B3] Aziz RK , Bartels D, Best AA, DeJongh M, Disz T, et al 2008. The RAST server: rapid annotations using subsystems technology. BMC Genomics. 9:75. 18261238 10.1186/1471-2164-9-75 PMC2265698

[jkab074-B4] Besemer J , Lomsadze A, Borodovsky M. 2001. GeneMarkS: a self-training method for prediction of gene starts in microbial genomes. Implications for finding sequence motifs in regulatory regions. Nucleic Acids Res. 29:2607–2618. 11410670 10.1093/nar/29.12.2607 PMC55746

[jkab074-B5] Camacho C , Coulouris G, Avagyan V, Ma N, Papadopoulos J, et al 2009. BLAST+: architecture and applications. BMC Bioinformatics. 10:421. 20003500 10.1186/1471-2105-10-421 PMC2803857

[jkab074-B6] Carrol D , Daszak P, Wolfe ND, Gao GF, Morel CM, et al 2018. The global virome project. Science. 359:872–874. 29472471 10.1126/science.aap7463

[jkab074-B7] Christie GE , Calendar R. 2016. Bacteriophage P2. Bacteriophage. 6:e1145782.doi: 10.1080/21597082.2016.1145782. 27144088 10.1080/21597081.2016.1145782 PMC4836473

[jkab074-B8] Davis JJ , Wattam AR, Aziz RK, Brettin T, Butler R, et al 2020. The PATRIC bioinformatics resource center: expanding data and analysis capabilities. Nucleic Acids Res. 48:D606–D612. 31667520 10.1093/nar/gkz943 PMC7145515

[jkab074-B9] Delcher AL , Bratke KA, Powers EC, Salzberg SL. 2007. Identifying bacterial genes and endosymbiont DNA with Glimmer. Bioinformatics. 23:673–679. 17237039 10.1093/bioinformatics/btm009 PMC2387122

[jkab074-B10] Duplessis CA , Biswas B. 2020. A review of topical phage therapy for chronically infected wounds and preparations for a randomized adaptive clinical trial evaluating topical phage therapy in chronically infected diabetic foot ulcers. Antibiotics. 9:377. 10.3390/antibiotics9070377 PMC7400337 32635429

[jkab074-B11] Ecale Zhou CL , Malfatti S, Kimbrel J, Philipson C, McNair K, et al 2019. multiPhATE: bioinformatics pipeline for functional annotation of phage isolates. Bioinformatics. 35:4402–4404. 31086982 10.1093/bioinformatics/btz258 PMC6821344

[jkab074-B12] Furfaro LL , Payne MS, Chang BJ. 2018. Bacteriophage therapy: clinical trials and regulatory hurdles. Front Cell Infect Microbiol. 8:376. 30406049 10.3389/fcimb.2018.00376 PMC6205996

[jkab074-B13] Górski A , Międzybrodzki R, Węgrzyn G, Jończyk-Matysiak E, Borysowski J, et al 2020. Phage therapy: current status and perspectives. Med Res Rev. 40:459–463. 31062882 10.1002/med.21593

[jkab074-B14] Grazziotin AL , Koonin EV, Kristensen DM. 2017. Prokaryotic virus orthologous groups (pVOGs): a resource for comparative genomics and protein family annotation. Nucleic Acids Res. 45:D491–D498. 27789703 10.1093/nar/gkw975 PMC5210652

[jkab074-B15] Hyatt D , Chen G-L, LoCascio PF, Land ML, Larimer FW, et al 2010. Prodigal: prokaryotic gene recognition and translation initiation site identification. BMC Bioinformatics. 11:119. 20211023 10.1186/1471-2105-11-119 PMC2848648

[jkab074-B16] Johnson LS , Eddy SR, Portugaly E. 2010. Hidden Markov model speed heuristic and iterative HMM search procedure. BMC Bioinformatics. 11:431. 20718988 10.1186/1471-2105-11-431 PMC2931519

[jkab074-B17] Kieft K , Zhou Z, Anantharaman K. 2020. VIBRANT: automated recovery, annotation and curation of microbial viruses, and evolution of viral community function from genomic sequence. Microbiome. 8:90. 32522236 10.1186/s40168-020-00867-0 PMC7288430

[jkab074-B18] Laffy PW , Wood-Charlson EM, Turaev D, Weynberg KD, Botté ES, et al 2016. HoloVir: a workflow for investigating the diversity and function of viruses in invertebrate holobionts. Front Microbiol. 7:822. 27375564 10.3389/fmicb.2016.00822 PMC4899465

[jkab074-B19] Li P-E , Lo C-C, Anderson JJ, Davenport KW, Bishop-Lilly KA, et al 2017. Enabling the democratization of the genomics revolution with a fully integrated web-based bioinformatics platform. Nucleic Acids Res. 45:67–80. 27899609 10.1093/nar/gkw1027 PMC5224473

[jkab074-B20] Lombard V , Ramulu HG, Drula E, Coutinho PM, Henrissat B. 2014. The carbohydrate-active enzymes database (CAZy) in 2013. Nucleic Acids Res. 42:D490–D495. 24270786 10.1093/nar/gkt1178 PMC3965031

[jkab074-B21] Luong T , Salabarria A-C, Edwards RA, Roach DR. 2020. Standardized bacteriophage purification for personalized phage therapy. Nat Protoc. 15:2867–2890. 32709990 10.1038/s41596-020-0346-0

[jkab074-B22] McNair K , Zhou C, Dinsdale EA, Souza B, Edwards RA. 2019. PHANOTATE: a novel approach to gene identification in phage genomes. Bioinformatics. 35:4537–4542. 31329826 10.1093/bioinformatics/btz265 PMC6853651

[jkab074-B23] McNair K , Zhou CE, Souza B, Malfatti SA, Edwards R. 2021. Utilizing amino acid composition and entropy of potential open reading frames to identify protein-coding genes. Microorganisms. 9:129. 33429904 10.3390/microorganisms9010129 PMC7827183

[jkab074-B24] Philipson C , Voegtly L, Lueder M, Long K, Rice G, et al 2018. Characterizing phage genomes for therapeutic applications. Viruses. 10:188. 10.3390/v10040188 PMC5923482 29642590

[jkab074-B25] Pires DP , Costa AR, Pinto G, Meneses L, Azeredo J. 2020. Current challenges and future opportunities of phage therapy. FEMS Microbiol Rev. 44:684–700. 32472938 10.1093/femsre/fuaa017

[jkab074-B26] Reis-Cunha JL , Bartholomeu DC, Manson AL, Earl AM, Cerqueira GC. 2019. ProphET, prophage estimation tool: a stand-alone prophage sequence prediction tool with self-updating reference database. PLoS One. 14:e0223364. 31577829 10.1371/journal.pone.0223364 PMC6774505

[jkab074-B27] Russell DA , Hatfull GF. 2017. PhagesDB: the actinobacteriophage database. Bioinformatics. 33:784–786. 28365761 10.1093/bioinformatics/btw711 PMC5860397

[jkab074-B28] Seemann T. 2014. Prokka: rapid prokaryotic genome annotation. Bioinformatics. 30:2068–2069. 24642063 10.1093/bioinformatics/btu153

[jkab074-B29] Tanizawa Y , Fujisawa T, Arita M, Nakamura Y. 2019. Generating publication-ready prokaryotic genome annotations with DFAST. Methods Mol Biol. 1962:215–226. 31020563 10.1007/978-1-4939-9173-0_13

[jkab074-B30] Tkavc R , Matrosova VY, Grichenko OE, Gostinčar C, Volpe RP, et al 2017. Prospects for fungal bioremediation of acidic radioactive waste sites: characterization and genome sequence of *Rhodotorula taiwanensis* MD1149. Front Microbiol. 8:2528. 29375494 10.3389/fmicb.2017.02528 PMC5766836

[jkab074-B31] Voelker R. 2019. FDA approves bacteriophage trial. News from the Food and Drug Administration. J Am Med Assoc. 321:638.doi:10.1001/jama.2019.0510. 10.1001/jama.2019.0510 30778586

[jkab074-B32] Yang H , Yu J, Wei H. 2014. Engineered bacteriophage lysins as novel anti-infectives. Front Microbiol. 5:542. 25360133 10.3389/fmicb.2014.00542 PMC4199284

